# Analysis of Optical Diffraction Profiles Created by Phase-Modulating MEMS Micromirror Arrays

**DOI:** 10.3390/mi12080891

**Published:** 2021-07-28

**Authors:** Tarek Mohammad, Siyuan He, Ridha Ben Mrad

**Affiliations:** 1 Department of Mechanical and Industrial Engineering, University of Toronto, Toronto, ON M5S 3G8, Canada; rbenmrad@mie.utoronto.ca; 2 Department of Mechanical and Industrial Engineering, Ryerson University, Toronto, ON M5B 2K3, Canada; s2he@ryerson.ca

**Keywords:** diffractive optics, light steering, micromirror arrays, optical phase control, spatial light modulator, surface micromachining

## Abstract

This paper presents modeling and analysis of light diffraction and light-intensity modulation performed by an optical phased array (OPA) system based on metal-coated silicon micromirrors. The models can be used in the design process of a microelectromechanical system (MEMS)-based OPA device to predict its optical performance in terms of its field of view, response, angular resolution, and long-range transmission. Numerical results are derived using an extended model for the 1st-order diffracted light intensity modulation due to phase shift. The estimations of the optical characteristics are utilized in the designs of an OPA system capable of active phase modulation and an OPA system capable of array pitch tuning. Both designs are realized using the Multi-User MEMS Processes (PolyMUMPs) in which polysilicon is used as structural material for the MEMS-actuated mirrors. The experiments are performed to evaluate the optical performance of the prototypes. The tests show that the individually actuated micromirrors, which act as phase shifters, can transmit the most optical power along the 1st-order diffracted beam by actively changing their out-of-plane positions. In addition, the 1st-order diffracted beam with high optical intensity can be steered for distance measurement.

## 1. Introduction

Photonic integrated circuit (PIC)-based optical phased array (OPA) systems generally consist of photonic components such as optical splitters, waveguides, couplers, antennas, and thermo-optic-tuning-based phase shifters on silicon chips [1,2]. On the other hand, microelectromechanical system (MEMS)-based OPA systems [3] utilize silicon gratings or mirrors that can directly act as coherent emitters to obtain a relative phase difference of the pairing-diffracted light waves in the reflection mode. This leads to a relatively simple and straightforward design and construction of OPA systems. Compared with its silicon PIC counterpart, a MEMS based OPA offers less optical insertion loss and less power consumption [4], without compromising the fast response and high reliability. In silicon photonic-based OPA systems in which the refractive index changes with temperature, inefficient heat dissipation can cause temperature offsets and crosstalk between the adjacent phase shifters [5], leading to inaccurate phase shifting and device malfunction. To overcome the temperature-gradient effects, a proper heat dissipation through either modification of the structures or use of different materials that are compatible with post-fabrication processing of CMOS wafers is required [6,7]. On the other hand, the MEMS-based OPA systems provide a relative phase shift in free space through their highly reflective elements, mitigating the temperature-gradient effects. Therefore, the MEMS-grating or micromirror-based OPA systems to control the phase of light leading are emerging as practical implementations for phased array beam steering.

Many micromirror arrays were previously developed for a number of applications such as spectroscopy, optical switching, digital light-processing projectors, laser communication, and confocal microscopy [8,9]. Depending on their target applications, the motion types, actuation strokes, and operating speeds of those micromirror arrays were very different. For example, Texas Instrument’s digital micromirror device (DMD) [10] utilized a total of 62,500 micromirrors (with a size of 16 µm each) along an array of 250 × 250 to achieve an aperture size of 4 × 4 mm^2^. Milanovic et al. [11] designed an electrostatically actuated 4 × 4 tip-tilt-piston micromirror array in which each mirror size was 0.8 mm. The array demonstrated an optical angle of ±10° and a piston motion of ±24 µm at an applied voltage of 150 V. Due to their design requirements, the micromirrors required a long range of actuation strokes to modulate the optical phase by a magnitude of 2*π* radian and more, which led to high power requirements. Most of the previously designed conventional micromirror arrays are not suitable for high-speed laser-beam steering at wide field of view due to either the large mirror sizes or the large array pitch sizes. Grating light valves [12,13] driven by microactuators are capable of active phase shifting, but the optical elements usually have deformable membranes with rigid suspension features, leading to high driving-voltage requirements and wide laser-beam divergence angles. A few OPA systems based on low-mass reflective elements with soft actuating springs were recently reported in the literature [14,15,16]. However, the surface micromachining was challenging because the reflective elements were required to be made of narrow and tightly spaced suspended silicon microstructures with a high aspect ratio in the lateral dimensions. The OPA systems with few phase-shifting elements suffered from low scanning resolution. A large number of individually actuated phase shifters would be required for high-resolution laser steering in the active phase-modulation approach, leading to high complexity in control.

This paper presents the analytical modeling of diffracted light transmitted from OPA micromirrors. The optical diffraction model shows a relationship among the diffraction angle, array pitch, and laser wavelength. A standard model of the diffracted light-beam intensity is extended to estimate the light-intensity changes due to the phase shift performed by piston-type mirrors used in OPA systems. To validate the models, two key MEMS-micromirror-based OPA systems are prototyped using the standard PolyMUMPs [17,18]. The first device utilizes an array of piston-motion micromirrors that are individually actuated by electrostatic parallel plate microactuators. The second device utilizes a pair of lateral comb-drive actuators located at both sides of the micromirror array to generate the force required for in-plane motion of the micromirrors. The former OPA device realizes the active phase-modulation technique, whereas the latter device deploys the active pitch-modulation technique for beam steering.

## 2. Theory and Methods

After being reflected from OPA metal-coated silicon micromirrors, light waves propagate in the far field and create a diffraction profile by the constructive and destructive interferences of the waves from the periodic structural profiles created by a sequence of the mirror motions along the array. The optical performance of a MEMS-based OPA device is determined by its pitch, aperture, and number of phase shifters used in its array [4]. The 0th-order and 1st-order diffracted light beams are of particular interest in long-range scanning because the maximum radiation transmits along either of these two directions. Many classical textbooks on optics provide general models for optical diffraction, but they usually consider diffraction gratings that consist of multiple layers of alternating materials of varying refractive indexes to recreate the effect of a prism by which an output laser beam can be deflected. It is therefore important to develop a model for the higher-order diffracted light so that an accurate phase shift by OPA reflective elements can be practically achieved.

### 2.1. Analysis of Light Diffraction from Phase-Modulating Micromirror Array

**Figure 1:**
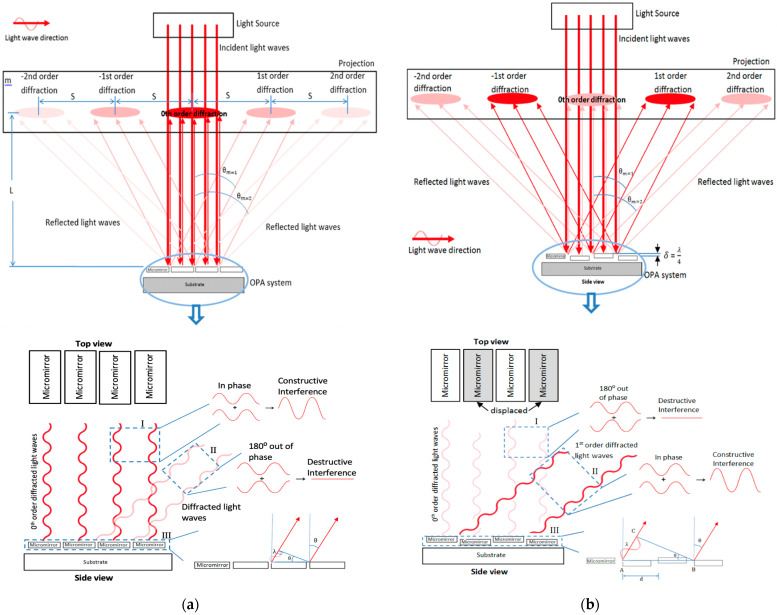
(**a**) Schematic of diffraction profile created by a microelectromechanical system (MEMS)-based optical phased array (OPA) system when illuminated during its unactuated state as incident light waves reflect from the mirror reflective surfaces and through gaps; (**b**) Schematic of illuminated the MEMS-based OPA system as every other suspended micromirror is pulled toward the substrate and the phase of the reflective light wave front is perturbed by setting up an interference field.

In this analysis, light is considered as monochromatic (no change in its wavelength) and coherent (light waves are in phase). Figure 1a shows the unactuated state of an OPA system upon which light waves are incident-normal to the reflective surfaces of its planar micromirrors. During this state, the reflected light waves are in phase (i.e., they have no path difference traveling back in straight lines) and there is a constructive interference between them. This results in a bright fringe at the center of the diffraction pattern on the projection screen, which is known as central maximum or 0th-order (order number *m* = 0) diffracted light. Partial constructive interferences can occur in the other pairs of output light waves that are deflected at certain angles *θ_m_* from normal to the reflective surface, leading to the additional bright fringes or higher-order diffracted lights. From Figure 1a, we can get *tanθ_m_* = *mS*/*L*. Since the distance of bright fringe centers *mS* is much smaller than the distance between the mirrors and projection *L*, we can approximate *sinθ_m_* ≈ *tanθ_m_* = *mS*/*L*. This approximation is valid for small angles (<30°). Due to this limitation, the range of scan angles achieved by most diffractive optics remain narrow.

There is an optical path-length difference Δ*L* between the deflected waves emerging from the mirror edges. From the triangle drawn in Figure 1b, we can get Δ*L = d·sinθm*, where *d* is the micromirror pitch by which the pairing waves are separated. When the path-length difference between these pairing waves is *λ*, they are in phase, and constructive interference occurs. As a result, bright fringes are observed on either side of the central maximum. Since the interference of the pairing waves is separated by the distance *d* and has a path-length difference equal to multiple of wavelengths, we can get *d·sinθm* = *mλ* by Bragg’s law [13]. On the other hand, when the path-length difference between the pairing waves is *λ*/2, they become out of phase (relative phase shift of *π* radian or 180°) and cancel each other. This applies to the pairing-deflected waves emerging from all the mirror edges. If the path-length difference is equal to the multiple of half wavelengths, we can get *d·sinθm* = (*m*·+·1/2) *λ* [19]. Therefore, there would be no resultant wave, which means no main lobe consisting of the diffracted light waves would be observed in the far field. During the actuated state of the OPA system, every other micromirror is moved out of plane to create a vertical displacement *δ* with respect to its neighboring unmoved micromirror along the array, as shown in Figure 1b. As a result, the light waves from these displaced micromirrors travel an extended distance and experience a delay with respect to the light waves from the neighboring unmoved micromirrors. This delay results in a relative phase shift between the pairing light waves. Since the light waves from the displaced mirrors travel a total additional distance of 2*δ* as compared to the other light waves from the neighboring mirror surfaces, the relative phase shift Δ*φ* of the waves can be given as (2*π*·2*δ*)/*λ* [20]. The relative displacement must be equal to a quarter of the laser wavelength *δ* = *λ*/4 to make the pairing-reflected light waves (traveling normal to the mirror surfaces) exactly out of phase (phase shift of *π* radian or 180°) and cancel each other (see Box I in Figure 1b). As a result, the main lobe consists of the 0th-order diffracted light waves, and would not be observed on the projection screen.

From Box III in Figure 1b, we can get *sinθ* = *AC*/*AB*. The light wave from point A must be in phase with the light wave from point B, and this occurs when the path length difference AC is equal to one wavelength, which leads to phase shift of (2*π·AC*)/*λ* or 2*π* between the pairing waves traveling along the 1st-order diffracted light beams. In order to obtain the desired optical phase shift, the displacement of the mirrors in an OPA system can be configured into two groups: one group of mirrors is set to 0 radian phase and another group of mirrors is set to *π* radian phase. This is known as the binary phase-shift pattern [21]. From Box III in Figure 1b, AB is equal to the pitch between adjacent mirrors *d* times the number of mirrors used for one phase period 2*N*. Therefore, the half-angle $\theta_{m}$ of the diffracted laser beam by an OPA system can be expressed as:

(1) $$\theta_{m}=\pm {sin}^{-1}\left(\frac{m\lambda }{2Nd}\right)$$

Equation (1) is valid for the cases when the relative phase shift Δ*ϕ* between the adjacent mirrors is equal to *π* radian. The phase difference between these two neighboring light waves can be expressed as [13]:

(2) $$\Delta \phi =\frac{\pi }{\lambda }dsin\theta$$

To include the relationship between the relative phase shift and the diffraction angle, the equation can be rewritten as:

(3) $$\theta_{m}=\pm {sin}^{-1}\left(\frac{m\lambda \Delta \phi }{2N\pi d}\right)$$

Equation (3) shows that the diffraction angle is proportional to the laser wavelength and inversely proportional to the array pitch. Since the diffraction angle is dependent on the wavelength, the mirrors should be placed close to the light wavelength in magnitude [19]. In the diffraction profile, there are side lobes that represent radiation in undesired directions that are not usable in laser scanning. The side lobes can be minimized by reducing the number of interference orders in the far field [4]. This can be obtained by placing the micromirrors close to each other, which leads to a high fill-factor (reflective area/total area) of the array [21]. On the other hand, a pitch smaller than half of the laser wavelength is not desired due to potential optical coupling between adjacent shifters, which leads to a distortion in the diffraction pattern [4].

Since the OPA micromirrors generally require low actuation for creating the desired optical path difference (OPD), one of the commonly used microactuation techniques, such as electrostatic, electromagnetic, piezoelectric, and electrothermal actuators, can be deployed in the designs. Electrostatic microactuators are generally capable of translating microstructures precisely and rapidly over a stroke or travel range of few micrometers while maintaining a compact size. This actuation technique can be realized by silicon-based micromirrors, enabling an OPA system to obtain the necessary relative phase difference and to be operated at a low voltage for a range of laser wavelengths.

### 2.2. Optical Intensity Model for Diffracted Light Beams

**Figure 2:**
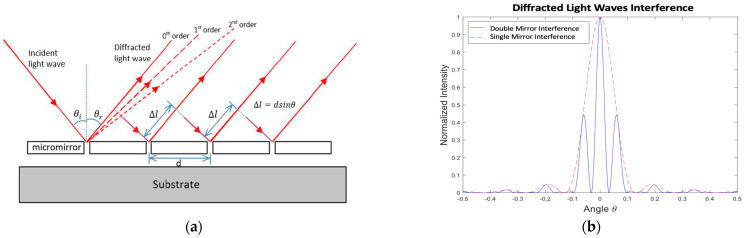
(**a**) Optical-intensity profiles of total diffraction patterns in variation with OPA micromirror width and pitch; (**b**) 1st-order diffracted light beam profile in variation with micromirror number in a MEMS-based OPA system.

Figure 2a shows that the parallel light waves with a monochromatic light wavelength *λ* are incident on the narrow micromirrors at an angle *θ*_i_ relative to the normal of the reflective surface. The mirrors are apart from each other by a planar distance *d*. The 0th-order diffracted light waves follow the specular reflection at an angle *θ*_r_ relative to the normal of the mirror surface, where *θ*_i_ = *θ*_r_. The light waves from all the mirrors interfere with each other. Therefore, the intensity of the resulting diffracted light pattern from an OPA system with multiple mirrors includes a diffraction factor due to the intramirror interference of light waves emerging within the same mirror, and an interference factor due to the intermirror interference of light waves emerging from the two adjacent mirrors [22]. The detail of the mathematical derivation is described in Appendix A. Incorporating both factors related to intramirror and intermirror interferences, the equations for the resulting light intensity along the 0th-order and 1st-order diffracted light beams can be respectively expressed as:

(4) $$I_{0\mathrm{th}}=I_{max}{\left(\frac{sin\alpha }{\alpha }\right)}^{2}cos^{2}\left(\frac{\phi }{2}\right)$$

(5) $$I_{1\mathrm{st}}=I_{max}{\left(\frac{sin\alpha }{\alpha }\right)}^{2}sin^{2}\left(\frac{\phi }{2}\right)$$

Equations (4) and (5) suggest that there is normally a phase difference between these two diffracted beams when all the planar OPA elements remain at the same height. Normally, constructive interference occurs along the 0th-order diffracted light waves (in phase) and destructive interference occurs along the 1st-order diffraction light waves (out of phase). When there is a height difference or optical path difference (OPD) among the OPA elements, we see a reverse effect occurring due to the relative phase difference applied to the diffracted light waves. Therefore, in order to switch the maximum radiation between the 0th-order and 1st-order diffracted light beams, the OPA reflective elements are usually required to have an optical path difference along the out-of-plane direction to realize an optical phase shift through selectively delaying or advancing the phase of the reflected light waves emerging from those relative to the phase of the reflected light waves emerging from adjacent optical elements.

#### Analysis of Light-Intensity Modulation due to Phase Shift

When an OPA system is illuminated, the portion of the incident laser beam that is reflected from the surface of the vertically displaced *δ* MEMS micromirror (piston-type) will travel a total distance of 2*δ* farther with respect to the remaining beam portion that is reflected from the surface of the adjacent MEMS micromirror with no displacement. This difference in out-of-plane distance between the elements realizes an OPD, causing a phase shift between the pairing light waves (see Figure 1b). When there is no relative vertical (out-of-plane) displacement of the micromirrors or no optical path difference (i.e., *δ* = 0), the phase difference between the neighboring light beam portions will be zero; i.e., *ϕ* = 2*π*/*λ* 2*δ* = 0. This results in maximum light intensity; i.e., $I_{0\mathrm{th}}=I_{max}cos^{2}\left(\frac{\phi }{2}\right)=I_{max}$ along the 0th-order diffraction direction. When the relative vertical displacement of the piston-type mirrors along the array becomes *δ* = *λ*/4, the phase difference of the reflected light waves becomes *ϕ* = *π*, which results in minimum light intensity; i.e., $I_{0\mathrm{th}}=I_{max}cos^{2}\left(\frac{\phi }{2}\right)=0$. This means no or minimum radiation along the 0th-order diffracted light will be observed on the projection screen due to the destructive interference between the light waves.

**Figure 3:**
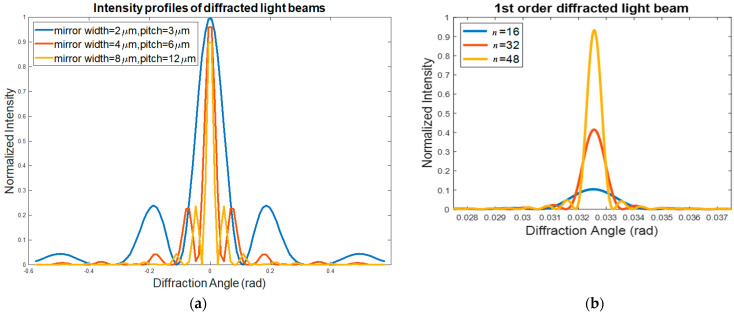
(**a**) Optical intensity profiles of total diffraction patterns in variation with OPA micromirror width and pitch; (**b**) 1st-order diffracted light beam profile in variation with micromirror number in OPA system.

At the same time, the applied phase difference of *π* will result in maximum light intensity along the 1st-order diffracted light beams; i.e., $I_{1\mathrm{st}}=I_{max}sin^{2}\left(\frac{\phi }{2}\right)=I_{max}$. This means the 1st-order diffracted light will be brighter and more intense. In this case, the laser beam spot along the 1st-order direction will appear on the projection screen. This can be later utilized for laser-beam steering. Equation (4) can be rewritten as:

(6) $$I=I_{max}{\left(\frac{sin\left(\frac{\pi a}{\lambda }sin\theta \right)}{\frac{\pi a}{\lambda }sin\theta }\right)}^{2}cos^{2}\left(\frac{\pi d}{\lambda }sin\theta \right)$$

where $I_{max}$, *a*, and *d* represent the maximum intensity, micromirror width, and pitch size, respectively. The change in the locations of the peaks in the diffraction patterns occurs due to the micromirrors’ width and pitch size variation (see Figure 3a). As the array pitch size increases, the angular positions at which the 1st-order diffracted light beams are located become closer to the unchanged position of the 0th-order diffracted light beams. Thus, a small pitch size enables a wide field of view (FoV). In addition, this variation in the micromirrors’ width and pitch size does not have a noticeable effect on the intensity of the peaks, as shown in Figure 3a. Similarly, Equation (5), representing light intensity along the 1st-order diffracted light beam, can be rewritten. The optical intensity of the 1st-order diffraction from an OPA system consisting of *n* micromirrors of width *a* (=*d*/2) can be obtained as:

(7) $$I_{1\mathrm{st}}=I_{max}{\left(\frac{sin\left(\frac{\pi d}{2\lambda }sin\theta \right)}{\frac{\pi d}{2\lambda }sin\theta }\right)}^{2}\frac{sin^{2}\left(\frac{n\pi d}{\lambda }sin\theta \right)}{sin^{2}\left(\frac{\pi d}{\lambda }sin\theta \right)}$$

The simulation results generated using Equation (7) are illustrated in Figure 3b, which shows that the locations of the peaks are not dependent on the number of micromirrors *n*. However, the peaks become sharper and more intense with an increase in the number of micromirrors along the array. In addition, the peak widths are found to be inversely proportional to the array size. As a result, a large number of micromirrors can result in sharp and intense laser spots, leading to a long-range transmission of radiation. It is therefore important to have an OPA aperture size that is equal to the laser-beam spot size in order to transmit the output laser beam at long distances which leads to higher optical performance by the OPA system. A relatively large optical aperture or array size of an OPA system can be achieved by increasing its reflective element number on the same plane. The above analysis thus provides useful insights into the optical design (i.e., mirror number, mirror size, array pitch, optical aperture, etc.) for the OPA systems in which a group of diffractive micromirrors work together to act as optical phase shifters with various periodic profiles.

## 3. Results and Discussion

### 3.1. Microelectromechanical System (MEMS)-Based Optical Phased Array (OPA) Design and Fabrication Considerations

A MEMS-based OPA device structure with a high resonant frequency requires its reflective elements (micromirrors or gratings) to be tiny, thin, and lightweight. The OPA micromirrors are required to be rapidly moved out of the plane in the sub-wavelength range (<1 μm) in order to obtain a desired optical phase shift. In addition, the width of the planar OPA micromirrors is required to be narrow in order to obtain a fine pitch of the array. The mechanical spring width of the micromirrors is also required to be proportionally narrow. A high fill factor (ratio of reflective area to total area) of a micromirror array leads to suppression of unwanted side lodes and to a high-power-output diffracted light beam or main lobe [21]. To maintain a high fill factor, the planar gap between the micromirrors should be minimized. Therefore, it is desired to place the microactuators beneath the mirror plates. This enables an increased reflective area of the array. One of the drawbacks of using reflective metal-coated silicon micromirrors is residual stress-induced curvature, which can affect planarity of the mirrors [23]. The midpoint deflection of a curved mirror must be small compared to the laser wavelengths to suppress any unwanted relative phase shift [17].

**Figure 4:**
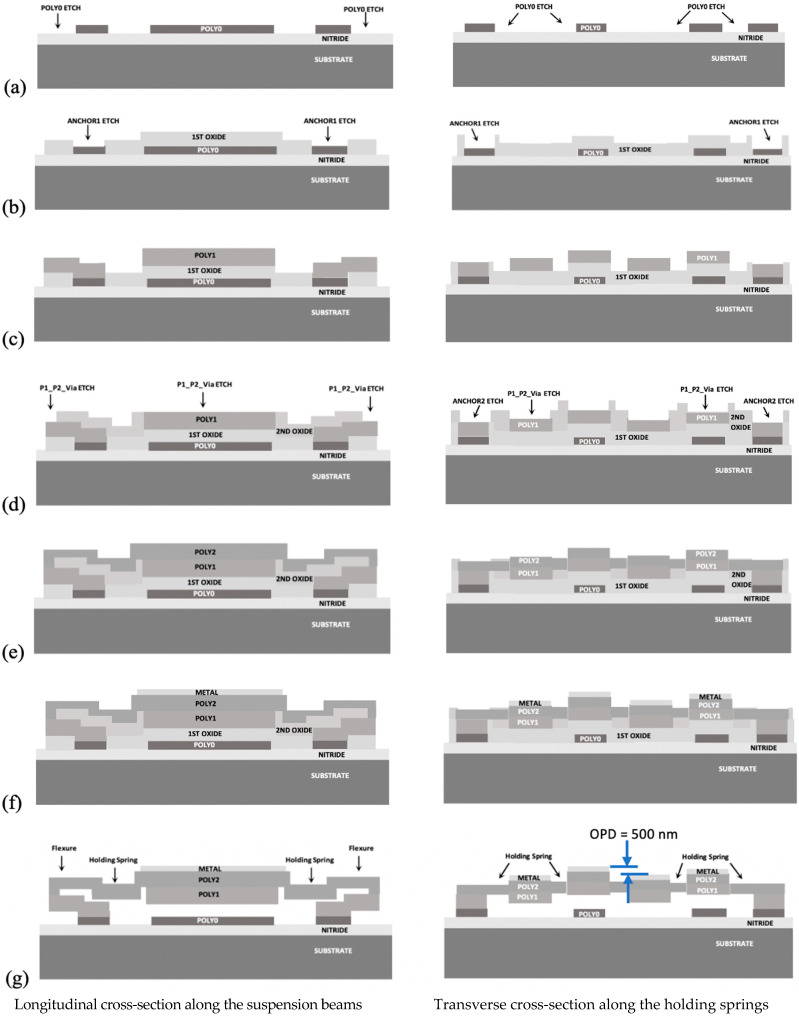
Schematic diagram showing details of the fabrication process for the pitch-tuning OPA micromirrors: (**a**) doped polysilicon bottom electrodes and GND (ground) layer; (**b**) bottom sacrificial oxide layer and DRIE of features; (**c**) second polysilicon layer for bottom parts of the mirror beam and the folded-beam flexure structures; (**d**) top sacrificial oxide layer and DRIE of features; (**e**) third polysilicon layer for the remaining parts of the mirror beams and spring structures; (**f**) gold metallization layer for the top mirror reflective surface; (**g**) HF release resulting in the free-standing structure.

To meet the above design requirements, a micromachining process with multiple structural layers is required. The critical dimension of a fabrication process determines the minimum allowable clearance and width of the micromirror structures (see Table 1). Such fine features and complex structures can be realized by using a surface micromachining process consisting of several polysilicon thin films (see Figure 4). Two OPA systems were fabricated using the same conventional surface micromachining process (PolyMUMPs). The process utilized a gold metallization layer with a thin chrome adhesion layer, which could be used to realize high optical reflectivity (~98%) of the polysilicon mirrors at a 650 nm laser wavelength and electrical conductivity of the electrostatic actuators through bond pads [17,18].

#### 3.1.1. Active Optical Phased Array Device

**Figure 5:**
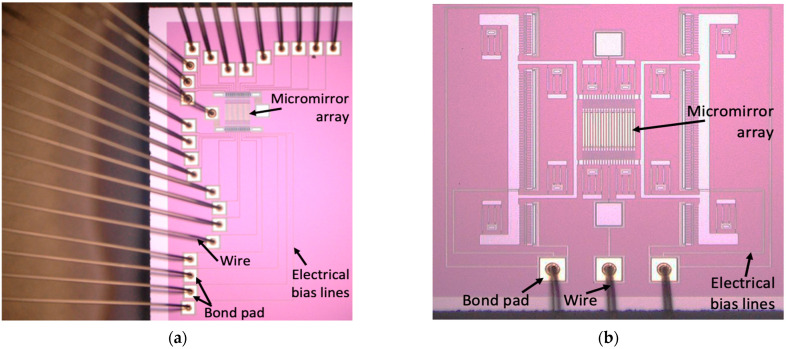
Microscopic images showing ball-stitch wire bonding used for (**a**) piston-motion micromirror-based OPA; (**b**) pitch-tunable micromirror-based OPA.

The first OPA device utilized an array of tightly spaced piston-motion micromirrors with polysilicon structures of high aspect ratio in the lateral dimensions. This approach enabled the realization of an electrostatic parallel plate actuator for each narrow and tiny micromirror, where the micromirror beam was suspended by actuating serpentine springs and an electrical interconnect line was routed underneath the micromirror to fit a tight pitch of the array (see Figure 5a). A vertical capacitor gap between each electrode pair of the electrostatic parallel plate actuators used in the OPA system was reduced by using the general dimple hole cuts available in the PolyMUMPs. This arrangement enabled the microactuators to be operated at a low voltage (56 V) and to generate a net displacement of 0.16 µm [17]. An electrical crosstalk between the adjacent micromirrors along the fine-pitched array was reduced by simply maintaining a relatively large planar gap or clearance between the micromirrors in compliance with the fabrication design rules. This configuration eliminated the need for protective sidewalls often used between tightly spaced OPA reflective elements in order to reduce electrical and mechanical crosstalks.

#### 3.1.2. Pitch-Tuning Optical Phased Array Device

A new pitch-tunable OPA was also designed in which all micromirrors were moved laterally to proportionally vary the scan angle for optical beam steering with a high angular resolution. The configuration allows all the micromirrors to be physically connected by holding springs that are electrically grounded, leading to an elimination of the electrical crosstalk between the adjacent micromirrors. The fabrication process flow is illustrated in Figure 4. An out-of-plane height difference between the adjacent mirrors required for the relative phase shift was achieved during the fabrication (see Figure 4g). This arrangement enabled an optical path difference at zero bias and eliminated the need for additional microactuators for an out-of-plane displacement of the phase shifters. A pair of electrostatic lateral comb-drive actuators was implemented in conjunction with mirror-positioned folded-beam flexures to enable accurate, fast, and guided motion, leading to a simplified design of the pitch-tunable OPA system (see Figure 5b). The comb-drive actuators were made of a single conductive layer in which the comb-drive fingers had varying thickness in order to maintain the minimum allowable planar gap or clearance between the fingers. This configuration enabled the surface micromachined comb-drive actuators to provide a relatively high actuation force required for the pitch tuning. The relation between the scan angle increment $\Delta \theta_{m}$ and the pitch variation Δ*d* was obtained by taking a derivation of the diffraction angle function given in Equation (3), which can be expressed as

(8) $$\Delta \theta_{m}=-\left(m\lambda \Delta \phi /2N\pi d^{2}\right)\Delta d$$

where *m* is the diffraction order and *N* is the number of elevated mirrors. In the proposed pitch-tuning OPA design, Δ*ϕ* and *N* remain constants during the lateral-only mirror motion induced by the comb drives from both sides.

A die consisting of both types of OPA devices was diced from the wafer and integrated using a standard ceramic PGA 144 pin 15 × 15 package. The thermo-sonic ball-stitch bonding process was utilized for the integration of both OPA types through their bond pads [24]. The packaged OPA system was interfaced with control electronics for beam steering. In addition, the reverse loop shape was implemented to maintain a compact size of the package. In the piston-type micromirror-based OPA prototype, all twenty-four (24) mirrors were individually actuated by corresponding parallel plate actuators. As a result, 24 corresponding electrical interconnect lines originated from the individual bottom electrodes of the micromirrors, then were routed through the individual tunnels and finally connected to the separate larger bond pads, which were located away from the micromirrors (see Figure 5a). The number of electrical bias lines used in the pitch-tunable micromirror-based OPA device was only three (3) because the OPA type did not require its micromirrors to be individually actuated (see Figure 5b). This led to a reduced complexity in control of the microactuators used in the device.

### 3.2. Experimental Assessment of OPA Devices

**Figure 6:**
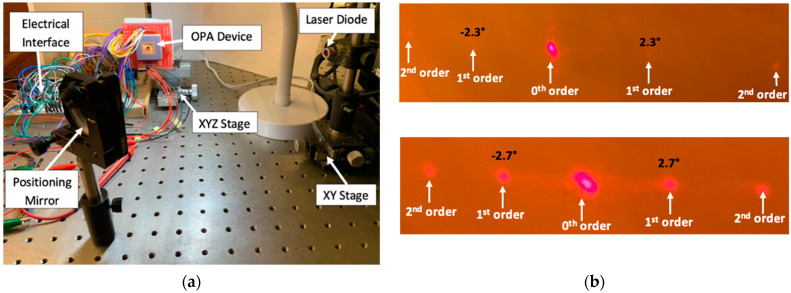
(**a**) Image of the experimental setup used to generate diffraction profiles and laser steering; (**b**) comparison of diffraction profiles created by active OPA (**top**) and pitch-tuning OPA (**bottom**).

Figure 6a shows the experimental setup for evaluating the optical performance by both OPA devices based on the piston-motion micromirrors and the pitch-tunable micromirrors. A laser diode was used to project a laser beam (spot size of 3 mm) on a mounted mirror surface. The positioning mirror was used to locate and redirect the incident laser beam on the reflective surface of the OPA device. The optical alignment could be made by using positioning stages in order to focus the laser beam on the target OPA device. The output laser beam emitting from the OPA reflective surface could be observed on a projection screen. The voltage input to the OPA device was provided by a signal-function generator and a power amplifier. Thus, the out-of-plane displacement of the piston-type micromirrors in the OPA device could be driven to activate and steer higher-order diffracted light beams on the projection.

**Figure 7:**
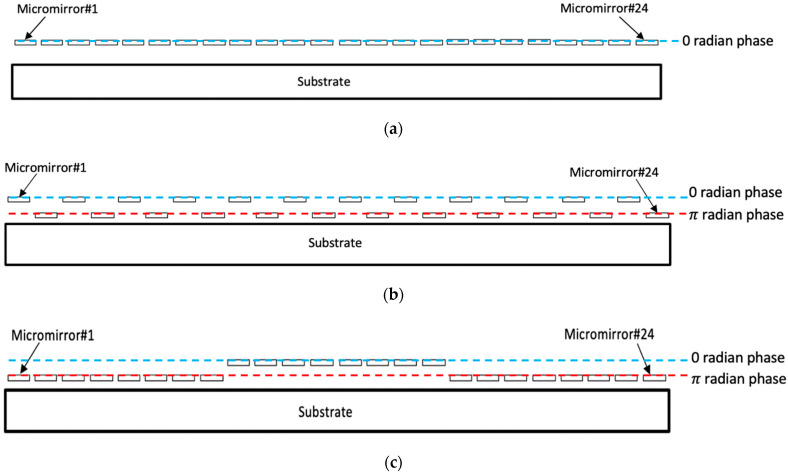
Schematics of various patterns of binary phase shift created by the piston-type OPA device consisting of 24 micromirrors with: (**a**) no phase shift; (**b**) a number of displaced micromirrors per phase period of 1; (**c**) a number of displaced mirrors per phase period of 8.

In an OPA-based scanner, the 1st-order diffracted light beam is usually used for laser scanning because it will be deflected at a certain diffraction angle with respect to the central axis. On the other hand, the 0th-order diffracted light beam remains undeflected, regardless of changes in the pitch size or in the binary phase-shift patterns along the OPA micromirrors. The magnitude of the diffraction angle along a higher order diffracted light beam can be modulated by bringing a variety of periodic profiles consisting of a binary phase-shift pattern. This can be achieved by changing the number of micromirrors used to create one phase period in the phase-shift patterns (see Figure 7). The specific periodic profile with a binary phase-shift pattern shown in Figure 7b caused the OPA device to generate its maximum scan angle. Eight (8) micromirrors were displaced per phase period to create the phase-shift pattern shown in Figure 7c.

**Figure 8:**
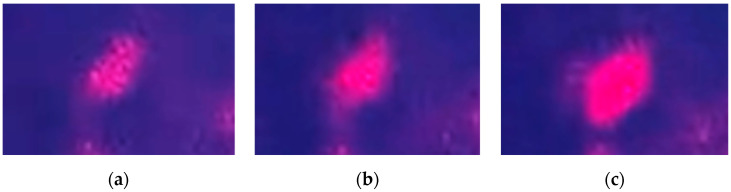
Far-field images of (**a**) higher-order diffracted light beam spot from the OPA during unactuated state; (**b**) the diffracted light with an increased intensity on the same spot during transition to actuated state; (**c**) the diffracted light with maximum intensity on the same spot due to the actuation.

Figure 8 shows a variation in the light intensity along a higher-order diffracted light beam on the projection due to the phase shift performed by the micromirrors out-of-plane motion. Figure 8a shows the initial diffracted beam spot or main lobe in an unactuated state of the piston-type micromirror-based OPA device. Figure 8b shows an increase of light intensity along that diffracted beam lobe during an actuated state where every other micromirror is actuated. When the amplitude of the micromirror displacement was a quarter of the laser wavelength, the light intensity reached the maximum along that direction (see Figure 8c). This was because of the constructive interference occurring between the pairing light waves along the higher-order diffraction caused by the optical path difference among the micromirrors, which would agree with the concept shown in Figure 1b. The light waves reflected from the displaced OPA micromirrors and reached the projection screen with a phase difference with respect to the light waves from the unmoved OPA micromirrors. However, the light intensity modulation along the 0th-order diffracted lobe was difficult to observe because of the unwanted specular reflection of light, which was superpositioned on the diffraction profile. There was a planar gap between every two adjacent micromirrors in the OPA device, and a portion of the incident laser beam reached the bottom layer through the gap (3.5 µm in this design).

**Figure 9:**
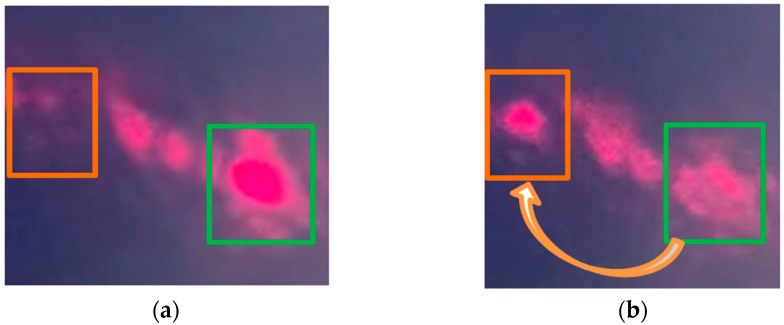
(**a**) Far-field image of a higher-order diffracted laser beam spot from the OPA during unactuated state; (**b**) far-field image showing a change of the laser beam spot position along the scan line caused by applying a different phase-shift pattern to the OPA micromirrors.

Figure 9 shows a diffracted beam spot changing its position on the projection screen due to the change of the binary phase-shift patterns. This caused the corresponding main lobes to be steered. Most radiation was observed along the 1st-order diffracted light beams. Through this test, it has been demonstrated that the scan angle of a higher diffracted light beam emitting from the OPA device can be changed. Therefore, the piston-motion micromirror-based OPA device can be used for optical steering.

The pitch-varying micromirror-based OPA device also was tested by using the same experimental setup. The control of this device was much easier, since the voltage was required to apply to the pair of lateral electrostatic comb-drive actuators only. In this OPA type, a binary phase-shift pattern, in which one group of mirrors is set to 0 radian phase and another group of mirrors is set to 3*π* radian phase, was already formed among the micromirrors during the fabrication steps (see Figure 4g). Thus, it created a strong diffraction profile at zero bias (see Figure 6b) where the maximum radiation was transmitted through the 1st-order diffracted light beam. A steering range of 0.06° was experimentally achieved by actively varying the array pitch at 32.5 $V_{pp}$ amplitude, which resulted in an angular resolution of 0.002° [18].

## 4. Conclusions

Analytical models of the light-diffraction profiles created by an array of micromirrors have been presented. It was shown that a fine pitch among the micromirrors can lead to a wide scan angle. During the design process, the models helped a designer to select the proper mirror size and the array pitch suitable for the application. Widely spaced micromirrors resulted in significant side lobes of the diffracted beams. The side lobes added noise or false positives in LiDAR scanning. To minimize the side lobes, a high fill-factor (reflective area/total area) of the array should be maintained. The fine-pitched micromirror arrays enabled relatively wide fields of view and high resonant frequencies. The OPA design parameters and the actuation requirements could be determined with confidence when the decisions were made based on the estimations using the models.

In the first device, the magnitude of the out-of-plane displacement by the phase shifters was equal to one-quarter of the laser wavelength used, which led to the desired optical phase shift of *π* radian. In the second device, the optical path difference was realized by slightly elevating every other micromirror along the array in order to form the required optical phase shift of 3*π* radian. Due to these preadjusted phase levels across the array, the required optical path difference was obtained at zero bias, and the design eliminated the need for additional microactuators for out-of-plane displacement of the micromirrors.

## Figures and Tables

**Table 1:** Design specifications for the Microelectromechanical System (MEMS)-Based Optical Phased Array (OPA) structures.

Design Parameters	Active OPA	Pitch-Tuning OPA
Micromirror width	4.5 µm	5 µm
Micromirror length	135 µm	4 µm
Micromirror beam length	4.25 µm	185 µm
Planar gap between adjacent mirrors	3.5 µm	5 µm
Number of micromirrors	24	20
Mirror pitch size	8 µm	7–10 µm (variable)
Maximum scan (half) angle at 635 nm wavelength	±2.3°	±2.7° to ±3.4°
Total array size (reflective surface area)	185 µm × 135 µm	195 µm × 180 µm
Fill factor	37.5%	40%–57%
Bond pad size	100 µm × 100 µm	100 µm × 100 µm
Number of bond pads	25	3 ^1^

^1^ Fewer electrical connections result in simplified control of the device.

## Appendix A

The optical intensity of the diffracted light profile is the product of functions representing intermirror and intramirror interferences (see Figure 2b). At first, the intermirror interference is considered, and an equation representing the interference factor due to light waves emerging from two adjacent mirrors is derived. The pairing reflected light waves can be in or out of phase when they reach a screen placed at a distance *L* from the mirror array, depending on the difference in the distances traveled. A reflected light wave on the projection screen can be expressed as [22]:

(A1) $$y_{1}=Asin\left(kL-\omega t\right)$$

where *A* is the amplitude of the signal, *ω* is the angular frequency, and *k* (=2*π/λ*) is the wave number. The reflected light wave emerging from the adjacent mirror travels an additional distance of ∆*l* (=*d·sinθ*) to reach to the screen, which can be expressed as:

(A2) $$y_{2}=Asin\left(k\left(L+dsin\theta \right)-\omega t\right)$$

These two light waves from the two neighboring mirrors can be summed up to model the total wave, as expressed by Equation (A3):

(A3) y=Asin(kL−ωt)+Asin(k(L+dsinθ)−ωt)

A combination of the intensity of the light waves at a projection point determines the amplitude of the total light waves. The amplitude of the total light waves can be obtained using Euler’s formula, which combines real *Re* and imaginary *Im* parts: $e^{i\theta }=\cos\theta +isin\theta$, where cosθ = *Re*$\left(e^{i\theta }\right)$ = $\frac{e^{i\theta }+e^{-i\theta }}{2}$ and sinθ = *Im*$\left(e^{i\theta }\right)$ = $\frac{e^{i\theta }-e^{-i\theta }}{2}$. Equation (A3) can be rewritten by considering the imaginary parts (sine components) for the total (complex) wave:

(A4) $$y=Ae^{i\left(kL-\omega t\right)}+Ae^{i\left(kL+kdsin\theta -\omega t\right)}=2A\cos\left(\phi /2\right)e^{i\left(kL+\phi /2-\omega t\right)}$$

The total amplitude along the diffracted light beam is the coefficient of the exponential term, which can be expressed as [20]:

(A5) $$A_{double}=2A\cos\left(\frac{\phi }{2}\right)$$

Since the intensity is proportional to the square of the amplitude [20], the above equation can be expressed in term of light intensity as:

(A6) $$I_{double}=I_{max}cos^{2}\left(\frac{\phi }{2}\right)$$

where the maximum intensity $I_{max}$ is the intensity of the total light waves at *θ* = 0; i.e., along the 0th-order diffracted light beam. Thus, the light intensity equation can be re-written as:

(A7) $$I_{double}=I_{max}cos^{2}\left(\frac{\pi dsin\theta }{\lambda }\right)$$

Normally, the reflective surfaces of the micromirrors along an array remain in one plane during unactuated condition, as shown in Figure 1a. Therefore, the edges of the OPA elements are not well exposed to the reflected light beam. In contrast to the 0th-order (*m* = 0) diffracted light beam; the 1st-order (*m* = 1) diffracted light beams emerging from the planar reflective surfaces would be minimum due to destructive interference. Therefore, as described in Section 2.1, the conditions for constructive and destructive interference between the pairing waves along the 0th-order and 1st-order diffractions are *d·sin*$\theta_{m}$ = *mλ* and *d·sin*$\theta_{m}$ = (*m* + 1/2) *λ*, respectively. Inserting the appropriate condition into Equation (A7) gives the following expression:

(A8) $$I_{double-1st}=I_{max}cos^{2}\left(\frac{\pi \left(1+\frac{1}{2}\right)\lambda }{\lambda }\right)$$

where the intensity $I_{double-1st}$ of the total light waves along the 1st-order diffracted light beam is zero. Therefore, the intensity equation for the 1st-order diffracted light beam can be written in a general form as:

(A9) $$I_{double-1st}=I_{max}sin^{2}\left(\frac{\phi }{2}\right)$$

Now the intramirror interference is considered, and an equation representing the diffraction factor due to light waves emerging within a single mirror of width a is derived. The wave signal can be expressed as [22]:

(A10) $$y=\frac{A}{a}sin\left(k\left(L+zsin\theta \right)-\omega t\right)$$

The total wave signal is the signal integrated over the entire width of the mirror (from *z* = 0 to *z* = *a*):

(A11) $$y=\int_{0}^{a}\frac{A}{a}sin\left(k\left(L+zsin\theta \right)-\omega t\right)dz$$

The phase difference between these two pairing light waves emerging from a single mirror reflective surface is expressed as [22]:

(A12) $$\alpha =\frac{1}{2}kasin\theta =\frac{\pi a}{\lambda }sin\theta$$

Putting Equation (A12) in the form of Euler’s formula and considering only the imaginary parts, the equation for the total wave signal becomes:

(A13) $$y=\int_{0}^{a}\frac{A}{a}e^{i\left(kL+kzsin\theta -\omega t\right)}dz=A\frac{sin\alpha }{\alpha }e^{i\left(kL+\alpha -\omega t\right)}$$

Since the intensity is proportional to the square of the amplitude [20], Equation (A13) can be used to deduce the intensity equation as:

(A14) $$I_{single}=I_{max}{\left(\frac{sin\alpha }{\alpha }\right)}^{2}.$$
